# Comprehensive machine learning analysis of a radiomics-based model for predicting microsatellite instability in right Colon Cancer

**DOI:** 10.3389/fonc.2026.1759980

**Published:** 2026-03-11

**Authors:** Junchuan Li, Li Liu, Xiaoqiong Zhong, Runxin Yang, Wenfeng Wang, Lian Yin, Dong Li, Hua Liu

**Affiliations:** Department of General Surgery, Yanjiang District People’s Hospital of Ziyang, Ziyang, Sichuan, China

**Keywords:** colorectal cancer, microsatellite instability, mismatch repair, prediction model, radiomics

## Abstract

**Objective:**

The objective of this study was to develop and validate a noninvasive radiomics-based machine learning (ML) model integrated with clinicopathological features for the prediction of microsatellite instability [deficient mismatch repair (dMMR)/microsatellite instability—high (MSI-H)] status in right colon cancer, aiming to provide a preoperative decision-making tool for clinical practice.

**Methods:**

A total of 247 patients with right colon cancer [43 dMMR and 204 proficient mismatch repair (pMMR)] who underwent radical resection between January 1, 2017, and 31 December 2024, were enrolled and randomly divided into a training set (70%) and a test set (30%). Preoperative contrast-enhanced computed tomography (CT) images were processed using 3D Slicer for region of interest (ROI) delineation and radiomics feature extraction. The intraclass correlation coefficient (ICC) was used to assess interobserver consistency, while the least absolute shrinkage and selection operator (LASSO) regression method was applied for feature selection. Logistic regression (LR), random forest (RF), support vector machine (SVM), and extreme gradient boosting (XGBoost) were used to construct radiomics models. The RF algorithm was selected to build a joint clinicopathological–radiomics model, and patients with left colon cancer served as the external validation set. Receiver operating characteristic (ROC) curves, calibration curves, and decision curve analysis (DCA) were used to evaluate diagnostic efficiency.

**Results:**

A total of 107 radiomics features were extracted, with 17 stable features retained after ICC filtering (ICC ≥ 0.75) and LASSO regression with 50% cross-validation. The RF algorithm outperformed other models in the radiomics model, with area under the curve (AUC) values of 0.98 in the training set and 0.96 in the test set. The joint model integrating the RF algorithm and clinicopathological variables (e.g., sex, age, tumor long diameter, histological type, pN, pM, pTNM, and differentiation degree) achieved the highest predictive performance, with AUC values of 0.99 (training set) and 0.97 (test set), which were significantly higher than those of the radiomics model and the clinical model alone. External validation with left colon cancer data also showed an AUC of 0.81, indicating good generalizability. The calibration curves demonstrated satisfactory probability prediction, and the DCA confirmed that the joint model provided greater clinical net benefit across the entire threshold probability range.

**Conclusion:**

The RF-based joint clinicopathological–radiomics model exhibited excellent performance in predicting the dMMR status in right colon cancer, with good generalizability across the entire colon. This noninvasive model can serve as a reliable clinical decision support tool to optimize risk stratification and guide early intervention for patients with right colon cancer.

## 1 Introduction

Traditional imaging modalities for tumor diagnosis rely primarily on physicians’ qualitative assessments of the lesion location, morphological characteristics, density profiles, and infiltration extent. This subjectivity-driven paradigm has long been plagued by interobserver variability, particularly when differentiating early-stage lesions from benign proliferative lesions or when evaluating subtle peritumoral infiltration. Recent advancements in radiomics have transformed this paradigm by enabling the extraction of high-dimensional, quantitative features from standard imaging modalities. Radiomics, a multidisciplinary field that combines medical imaging and artificial intelligence, offers a noninvasive, dynamic, and operator-independent analysis of intratumoral heterogeneity through computational algorithms (1). This approach converts radiographic images into actionable, mineable data, facilitating the development of predictive models that complement traditional diagnostic workflows (2–4). After feature extraction, machine learning (ML) techniques are used to construct predictive models. Common algorithms include support vector machine (SVM), artificial neural network (ANN), Bayesian network (BN), decision tree (DT), *k*-nearest neighbor (KNN), random forest (RF), extreme gradient boosting (XGBoost), and clustering methods (5–7). These algorithms enable the identification of complex patterns and associations within radiomics datasets, improving diagnostic accuracy and prognostic prediction in oncology. Through big data analytics, radiomics holds significant promise for personalized medicine applications, including treatment prediction, biomarker discovery, and patient stratification. In contemporary oncology research, ML has emerged as a powerful tool for the prediction of lymph node metastasis, treatment response, and prognosis (8–11). On the other hand, molecular immunology and genetic studies have increasingly focused on identifying the risk factors that drive tumorigenesis and metastatic progression (12–18). Although TNM remains the gold standard for prognosis assessment and treatment planning, tumor heterogeneity is increasingly attributed to the complexity of the tumor microenvironment and diversity of the immune cell components (19). In addition, deep learning (DL) algorithms, a subset of ML, can construct multilayer neural networks to provide more accurate predictive outcomes. Studies have confirmed that DL can effectively detect microsatellite instability [deficient mismatch repair (dMMR)/microsatellite instability—high (MSI-H)] in gastric and endometrial cancer (20, 21). MSI-SEER enables the prediction of MSI in gastric cancer and colorectal cancer (CRC) (22). A DL model based on H&E histological images has outperformed experienced pathologists in the prediction of MSI in CRC (23, 24). In the future, H&E-stained sections using the Deepath-MSI model could potentially obviate the need for routine immunohistochemistry and polymerase chain reaction (PCR) assays for MSI (25).

The present study introduces a novel approach by developing a preoperative radiomics-based predictive model, providing a noninvasive alternative to conventional tissue-based diagnostics. This methodology leverages the intrinsic correlation between imaging features and molecular phenotypes, with the potential to enable the early identification of dMMR tumors. Our objective was to establish a clinical decision support system to identify patients at risk for cancer and facilitate early intervention for these patients.

## 2 Data and methods

### 2.1 Research objective

Clinical data were collected from 247 patients with right colon cancer who underwent radical resection surgery at the Department of Gastrointestinal Surgery at Yanjiang District People’s Hospital of Ziyang and Ziyang Central Hospital between 1 January 2017 and 31 December 2024. The cohort included 43 patients with dMMR and 204 with proficient mismatch repair (pMMR). Patients were randomly divided into a training set and a test set at a 7:3 ratio. All procedures were performed in accordance with the Declaration of Helsinki and relevant regulatory guidelines. This retrospective study was approved by the Ethics Committee of Yanjiang District People’s Hospital of Ziyang. Written informed consent was waived due to the retrospective nature of the research.

### 2.2 Inclusion criteria

The inclusion criteria were as follows: 1) patients with complete sets of clinical, pathological, imaging, and immunohistochemical data; 2) patients with right colon cancer who received radical surgical treatment after CT scans; and 3) the MMR was assessed by postoperative pathology.

### 2.3 Exclusion criteria

We excluded the following: 1) patients who had undergone neoadjuvant chemotherapy, radiotherapy, or any biological therapy prior to surgical intervention; 2) patients who did not undergo surgical resection or those with unavailable or incomplete postoperative pathological datasets; 3) cases with CT images of suboptimal quality that rendered quantitative analysis unfeasible; and 4) patients whose target lesions were not discernible on preoperative CT imaging, precluding radiomics feature extraction and model construction.

### 2.4 Software selection

We used 3D Slicer for region of interest (ROI) delineation and texture feature extraction. The slicer-radiomics module within 3D Slicer was used to extract textures from the confirmed ROIs, including first-order statistics, shape-based features, gray-level co-occurrence matrix parameters, gray-level run-length matrix parameters, and neighborhood gray-tone difference matrix parameters from validated ROIs (1, 2).

### 2.5 Sketching of the primary lesion

**Figure 1:**
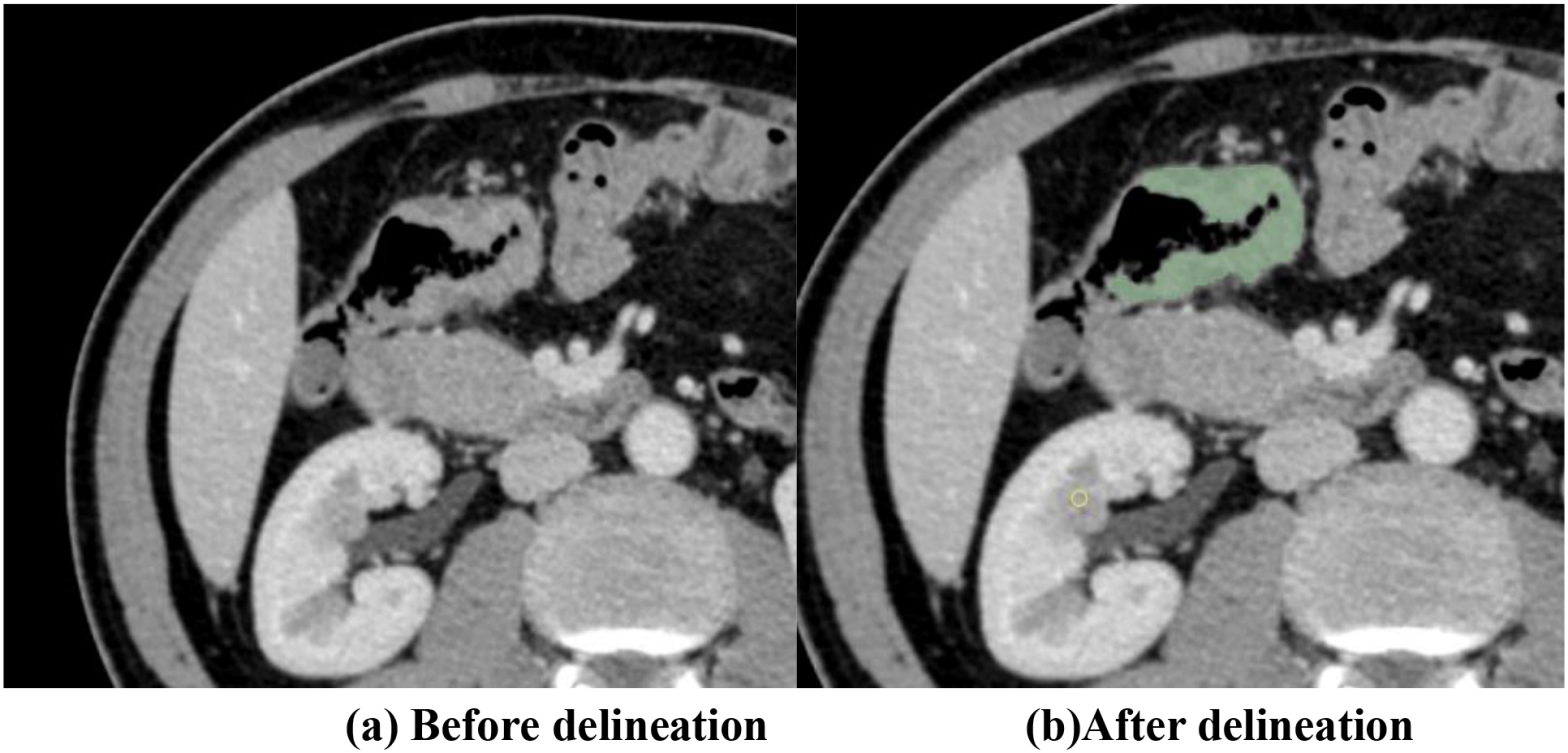
**(A)** Before delineation. **(B)** After delineation of the region of interest (ROI).

Contrast-enhanced CT DICOM datasets acquired 1–2 weeks preoperatively were imported into the 3D Slicer software. Patient-identifying information was removed prior to ROI delineation. Primary tumor ROIs were manually segmented layer-by-layer on axial slices by two radiologists with ≥3 years of abdominal imaging experience based on tumor attenuation characteristics and the anatomical continuity, with reference to multi-phase enhancement patterns (**Figure 1**) (3). To ensure interobserver consistency, the final segmentation results were independently reviewed and refined by two senior radiologists.

### 2.6 Repeatability of data

The texture features were independently extracted from the predefined ROIs on the CT images of 10 consecutive patients by two radiologists, the purpose of which was to assess intraobserver repeatability. The intraclass correlation coefficient (ICC) was calculated to evaluate interobserver consistency in the texture feature measurements. The ICC values were interpreted as follows: 0.81–1.00 (excellent consistency), 0.61–0.80 (good consistency), 0.41–0.60 (moderate consistency), 0.21–0.40 (fair consistency), and 0–0.20 (poor consistency).

### 2.7 Establishing the prediction model

Four ML models were used: logistic regression (LR), RF, SVM, and XGBoost.

### 2.8 Evaluating the predictive models

Receiver operating characteristic (ROC) curves were used to evaluate the predictive ability, with the area under the curve (AUC) and calibration curves employed for efficiency. In addition, decision curve analysis (DCA) was also performed to evaluate practicability (4).

### 2.9 Statistical methods

Categorical variables were analyzed with the chi-square test. Univariate LR was conducted to identify potential risk factors. Variables with a *p*-value <0.1 in the univariate analysis were included in the subsequent multivariate LR model to determine independent predictive factors. Feature selection, predictive model construction, statistical analyses, and data visualization were conducted using R software (version 4.0.4). Statistical significance was set at *p* < 0.05.

## 3 Results

### 3.1 General information

Consecutively collected patients, including 43 with dMMR and 204 with pMMR, were randomly divided into a training set and a test set at a ratio of 7:3. The training set consisted of 30 dMMR and 142 pMMR patients, while the test set included 13 dMMR and 62 pMMR patients. There were statistical consistencies in sex, age, BMI, location, long diameter, morphology, histological type, pT (pathological primary tumor), pN (pathological regional lymph node), pM (pathological distant metastasis), pTNM (pathological tumor, node, metastasis), intravascular cancer thrombus, nerve invasion, and differentiation (all from postoperative pathological diagnosis) between the training set and the test set (**Table 1**).

**Table 1:** Characteristics between the training set and the test set.

Characteristics		Training set		*p*	Test set		*p*
		dMMR (*n* = 30)	pMMR (*n* = 142)		dMMR (*n* = 13)	pMMR (*n* = 62)	
Sex	Male patients	16	78	0.873	6	34	0.568
	Female patients	14	64		7	28	
Age	<50 years	7	33	0.991	3	14	0.969
	≥50 years	23	109		10	48	
BMI	<18.5	3	14	0.881	1	3	0.914
	18.5–24	17	87		8	40	
	>24	10	41		4	19	
Location	Ileocecal	6	31	0.041	1	14	0.011
	Colon ascendens	14	57		6	25	
	Hepatic flexure	9	52		3	22	
	Transverse colon	1	2		3	1	
Long diameter	<5 cm	10	24	0.040	4	9	0.003
	≥5 cm	20	118		9	53	
Morphology	Ulcerative type	18	96	0.553	8	44	0.672
	Bulge type	12	44		5	17	
	Cauliflower type	0	0		0	0	
	Infiltrating type	0	2		0	1	
Histological type	Without mucus	16	131	<0.001	8	57	0.003
	Mucus or signet ring cell	14	11		5	5	
pT	T_1_	1	3	0.654	0	1	0.972
	T_2_	4	11		1	5	
	T_3_	20	94		9	41	
	T_4_	5	34		3	15	
pN	N_0_	22	48	<0.001	10	21	0.004
	N_+_	8	94		3	41	
pM	M_0_	27	135	0.281	12	59	0.677
	M_1_	3	7		1	3	
pTNM	I	3	13	0.222	2	6	0.448
	II	18	67		8	29	
	III	6	55		2	24	
	IV	3	7		1	3	
Intravascular cancer thrombus	No	24	108	0.642	10	47	0.932
	Yes	6	34		3	15	
Nerve invasion	No	21	88	0.407	9	36	0.455
	Yes	9	54		4	26	
Total		*n* = 30	*n* = 142		*n* = 13	*n* = 62	
Differentiated^a^	High	0	1	0.093	0	1	0.302
	High-medium	1	1		0	3	
	Medium	14	95		6	40	
	Medium-low	6	28		3	11	
	Low	6	10		3	4	
Total		*n* = 27	*n* = 135		*n* = 12	*n* = 59	

dMMR, deficient mismatch repair; pMMR, proficient mismatch repair; pT, pathological primary tumor; pN, pathological regional lymph node; pM, pathological distant metastasis.

a Missing pathology.

### 3.2 Screening and reducing dimensionality of the texture features

**Figure 2:**
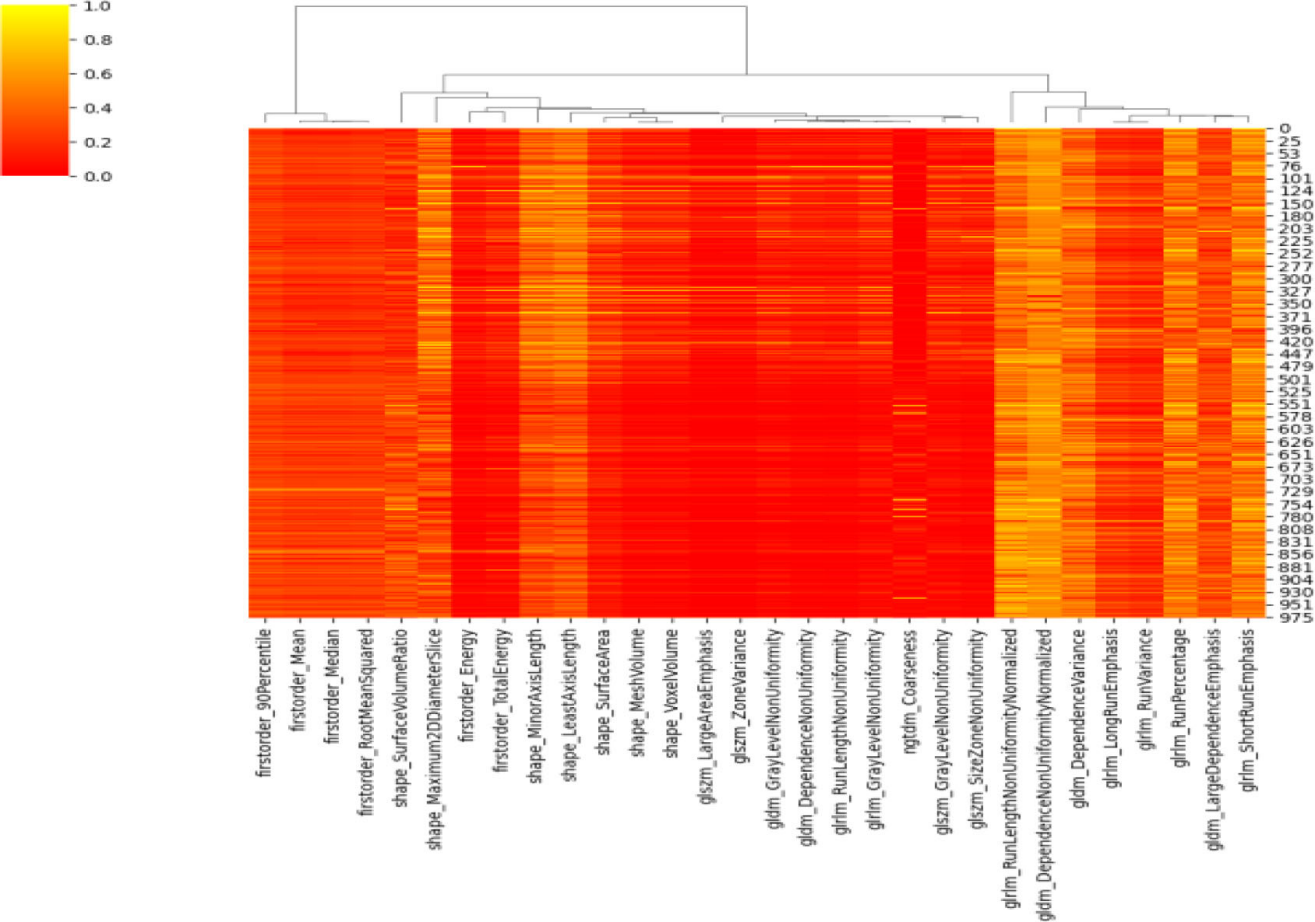
The 30 stable texture features.

**Figure 3:**
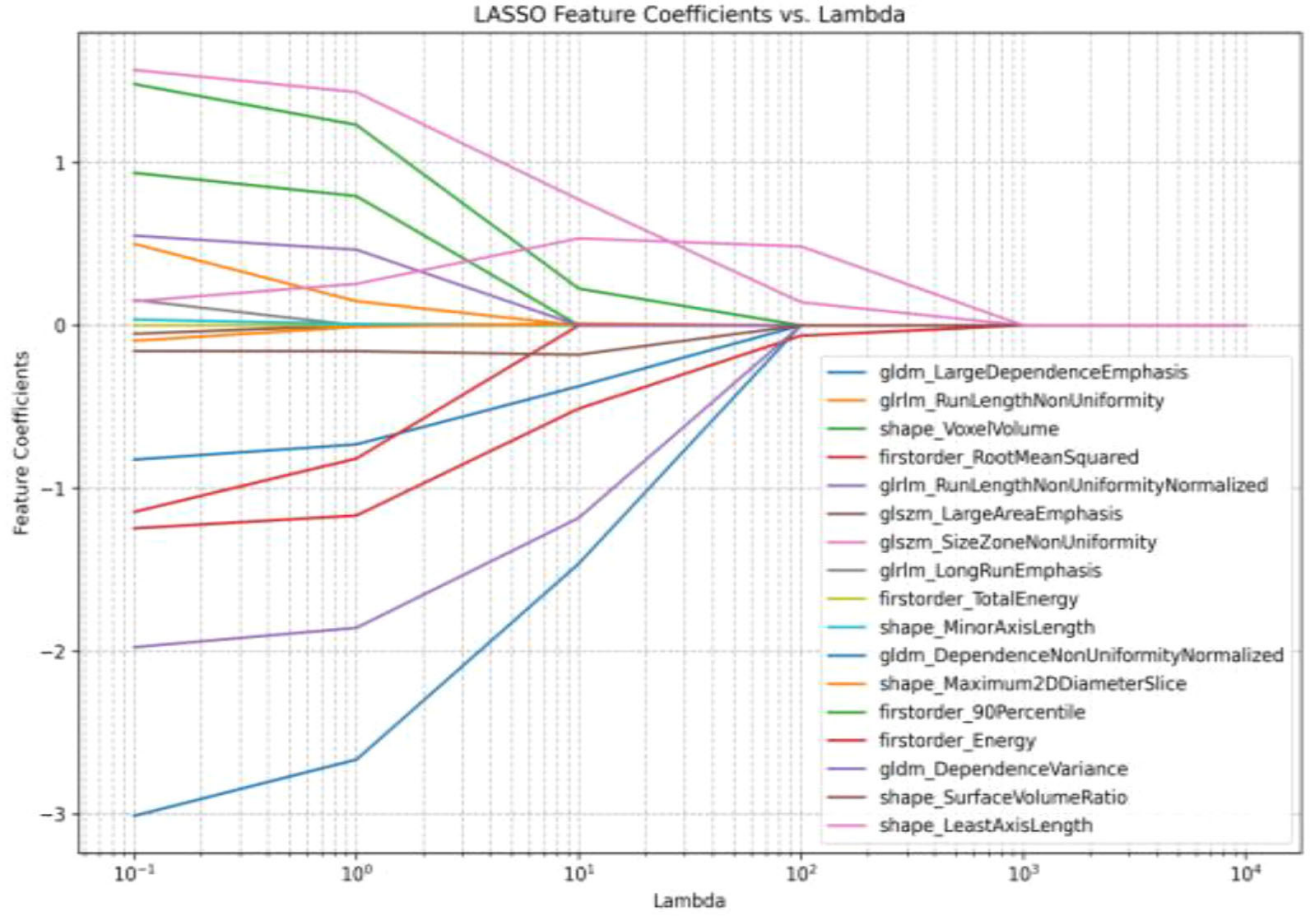
Least absolute shrinkage and selection operator (LASSO) regression of the 30 texture features.

**Figure 4:**
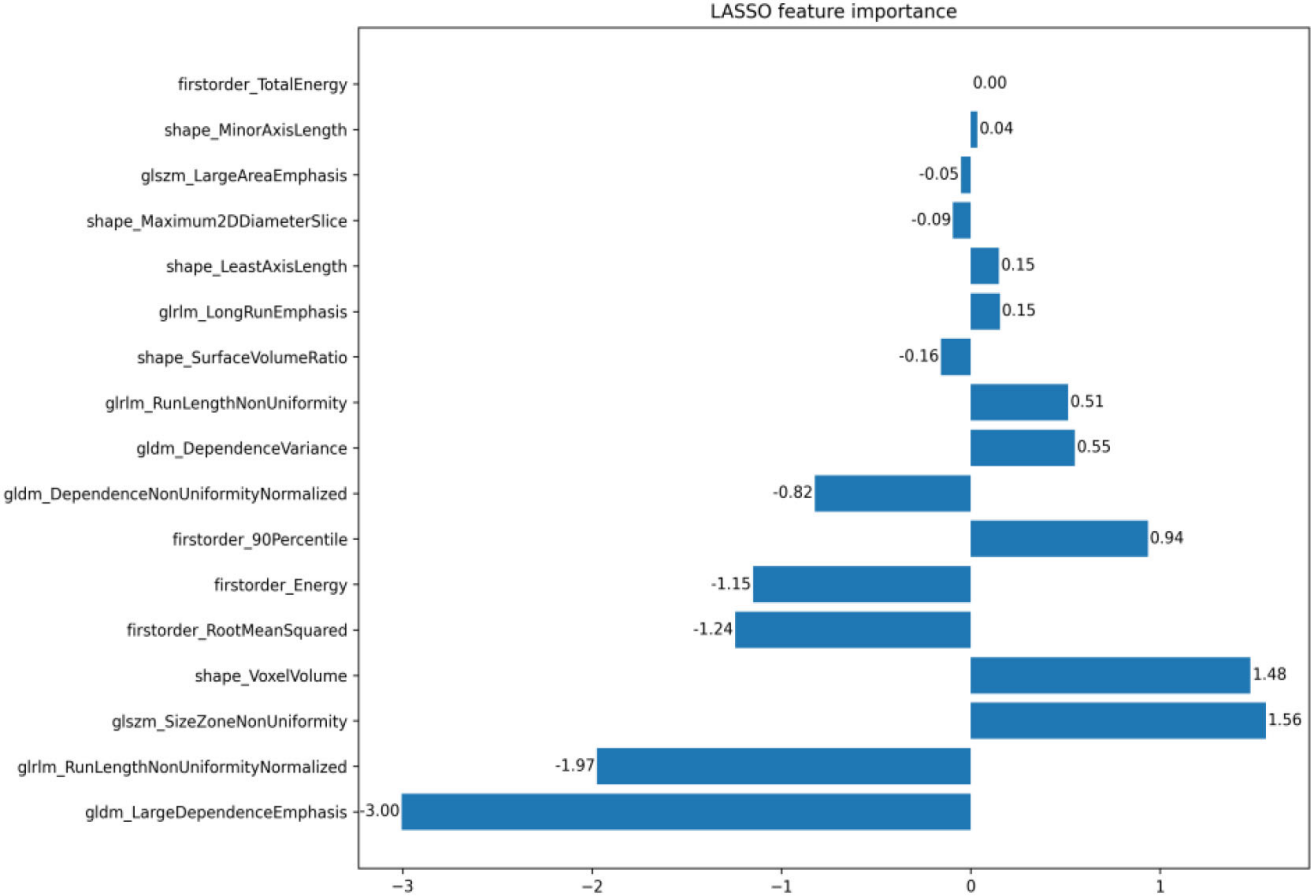
The 17 texture feature weight coefficients.

After extracting patients’ ROIs, a total of six categories encompassing 107 texture features were obtained (2). The ROIs from 10 randomly selected patients were independently evaluated by two physicians for ICC analysis. Subsequently, 77 features with ICC < 0.75 were excluded, yielding 30 stable features. Finally, the remaining 17 texture features and their weight coefficients were obtained using least absolute shrinkage and selection operator (LASSO) regression with 50% cross-validation (**Figures 2**–**4**).

### 3.3 Prediction model for dMMR right colon cancer by different ML methods

#### 3.3.1 Construction of the primary lesion radiomics prediction model

**Figure 5:**
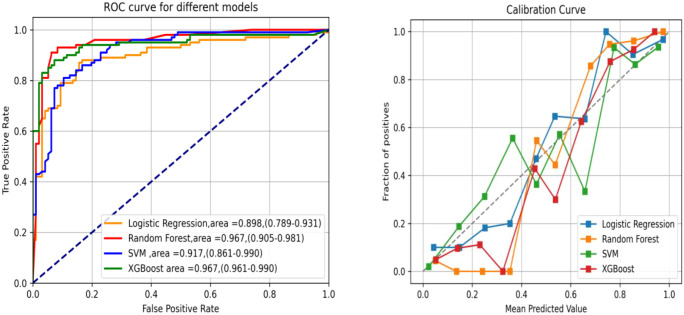
Receiver operating characteristic (ROC) and calibration curves of the primary lesion radiomics using four machine learning (ML) methods.

The AUC, accuracy, sensitivity, and specificity of LR, RF, SVM, and XGBoost in the primary lesion prediction model were calculated and compared in the training set and the test set (**Table 2**). The results revealed RF (AUC = 0.96) to be superior to the other algorithms in predicting dMMR. Calibration curves were drawn by repeated self-sampling 1,000 times, with all curves indicating good probability prediction (**Figure 5**).

**Table 2:** Deficient mismatch repair (dMMR) prediction model of the primary lesion radiomics by four machine learning methods.

Method		AUC (95%CI)	Accuracy	Sensitivity	Specificity
LR	Training set	0.91 (0.88–0.99)	0.94	0.85	0.86
	Test set	0.89 (0.78–0.93)	0.83	0.80	0.91
RF	Training set	0.98 (0.89–0.99)	0.98	0.93	0.91
	Test set	0.96 (0.91–0.98)	0.91	0.91	0.73
SVM	Training set	0.94 (0.83–0.98)	0.95	0.88	0.87
	Test set	0.91 (0.86–0.99)	0.84	0.82	0.93
XGBoost	Training set	0.97 (0.90–1.00)	0.99	0.92	0.85
	Test set	0.94 (0.96–0.99)	0.83	0.91	0.93

LR, logistic regression; RF, random forest; SVM, support vector machine; XGBoost, extreme gradient boosting.

#### 3.3.2 Construction of a joint clinicopathological–radiomics prediction model

**Figure 6:**
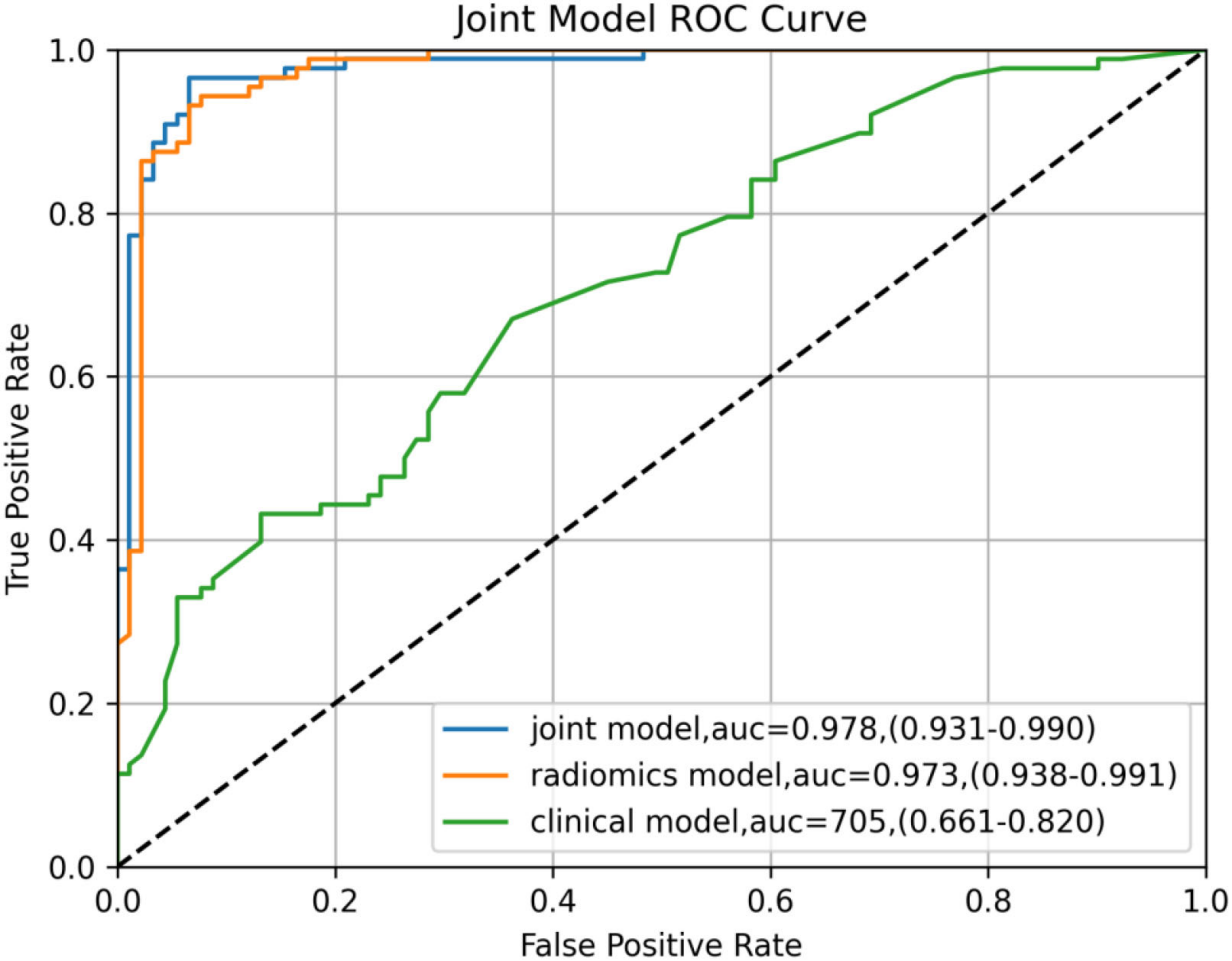
Receiver operating characteristic (ROC) curves of the different models for the right colon under random forest (RF) for predicting deficient mismatch repair (dMMR). .

**Figure 7:**
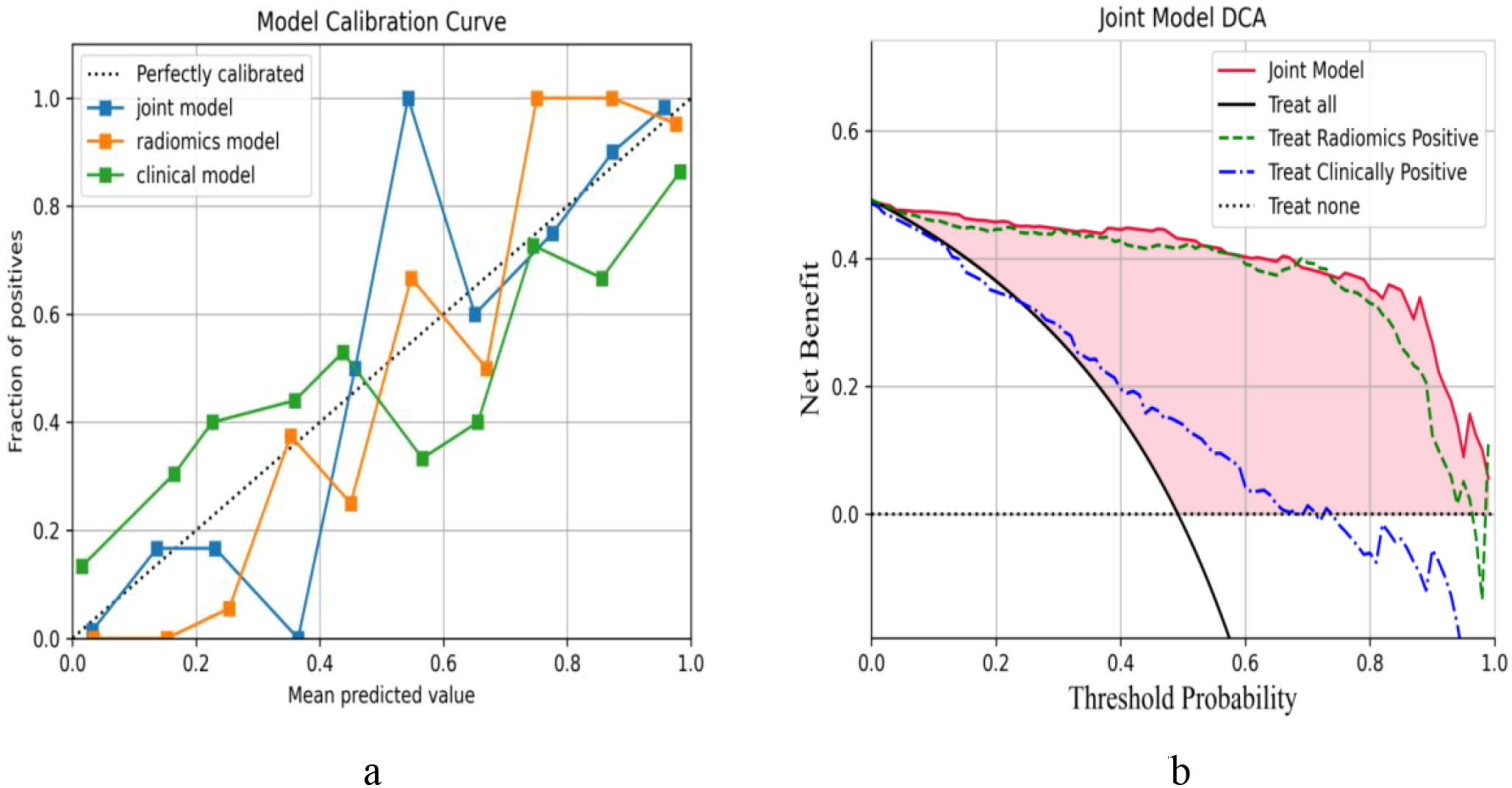
**(A, B)** Calibration curve and decision curve analysis (DCA) of the different models for the right colon under random forest (RF) for predicting deficient mismatch repair (dMMR).

The highest AUC with the RF algorithm was finally selected. For the clinical model, the clinicopathological variables identified as independent predictors via multivariate analysis—including sex, age, tumor long diameter, histological type, pN, pM, pTNM, and differentiation degree—were incorporated. The joint model was established based on the primary lesion after taking out a test set and performing 50% cross-validation on the remaining data. In the radiomics model, the AUC, accuracy, sensitivity, and specificity were 0.99 (95%CI = 0.98–0.99), 0.98, 0.97, and 0.96 in the training set and were 0.97 (95%CI = 0.93–0.99), 092, 0.94, and 0.91 in the test set, respectively. For the clinical models (established based on selected indicators such as sex, age, long diameter, histological type, pN, pM, pTNM, and differentiation), the training set values were 0.81 (95%CI = 0.69–0.86), 0.80, 0.60, and 0.80, while those of the test set were 0.70 (95%CI = 0.66–0.82), 0.61, 0.51, and 0.73, respectively. For the joint models (the 17 texture features in RF and the selected indicators mentioned above), the values were 0.99 (95%CI = 0.98–1.00), 0.99, 0.98, and 0.97 in the training set and were 0.97 (95%CI = 0.93–0.99), 0.93, 0.94, and 0.93 in the test set, respectively (**Table 3**). Notably, the joint model achieved the highest AUC of 0.978, which was significantly higher than that of the radiomics model and the clinical model alone, indicating that the new joint model would improve the prediction efficiency with RF (**Figure 6**). The calibration curve showed that the joint model had a better predictive probability, and DCA evaluated the influence of the model on clinical decisions (**Figures 7A, B**). Throughout the entire threshold probability range, the benefit of the joint model was better than that of the radiomics or the clinical prediction model.

**Table 3:** Prediction models with the random forest method.

Model		AUC (95%CI)	Accuracy	Sensitivity	Specificity
Radiomics model	Training set	0.99 (0.98–0.99)	0.98	0.97	0.96
	Test set	0.97 (0.93–0.99)	0.92	0.94	0.91
Clinical model	Training set	0.81 (0.69–0.86)	0.80	0.60	0.80
	Test set	0.70 (0.66–0.82)	0.61	0.51	0.73
Joint model	Training set	0.99 (0.98–1.00)	0.99	0.98	0.97
	Test set	0.97 (0.93–0.99)	0.93	0.94	0.93

AUC, area under the curve.

**Figure 8:**
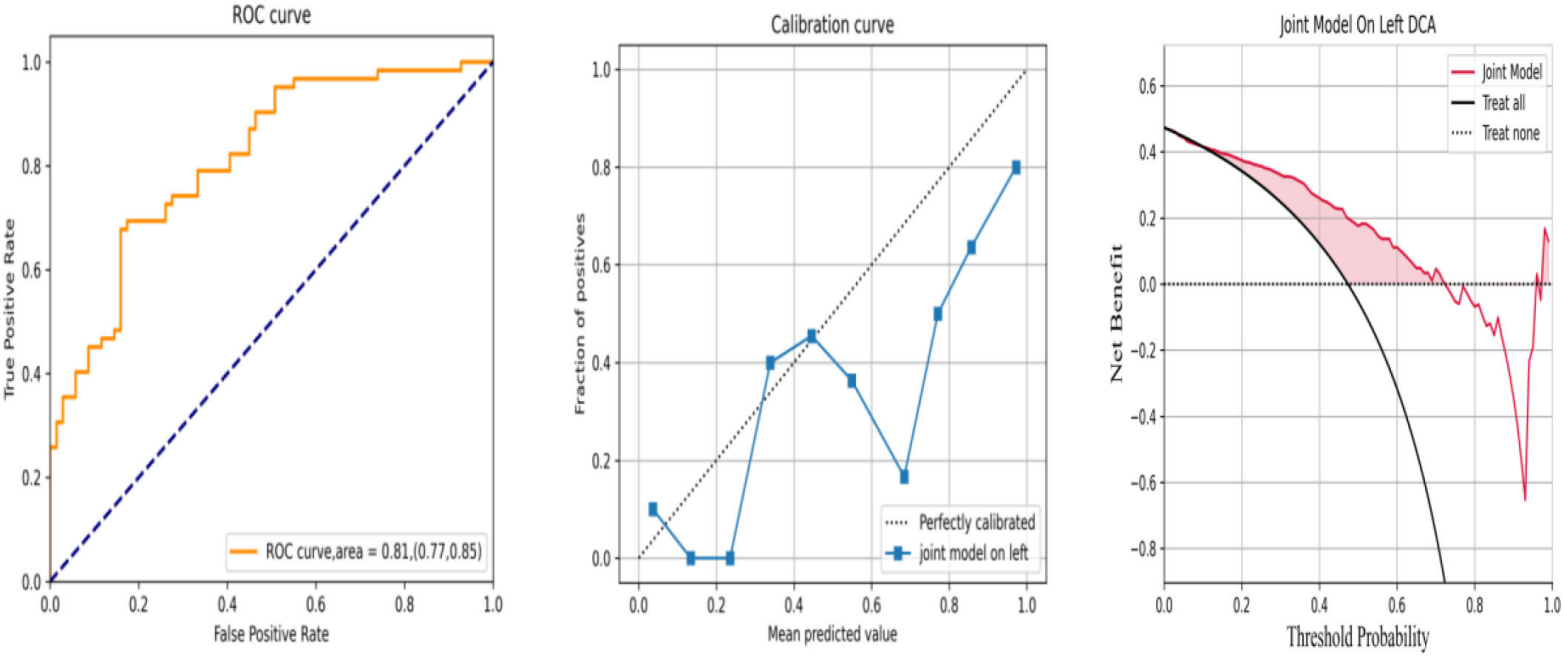
Receiver operating characteristic (ROC), calibration curve, and decision curve analysis (DCA) of the joint model in the left colon under random forest (RF) for predicting deficient mismatch repair (dMMR).

To further validate the effect of the joint model, data from the left colon were randomly selected as an external validation set to evaluate the generality and extrapolation ability. No statistically significant differences were observed between the training set and the external validation set (**Table 4**). As the RF algorithm achieved the best effect in the model, it was performed using the same method, achieving AUC, accuracy, sensitivity, and specificity values of 0.81 (95%CI = 0.77–0.85), 0.70, 0.85, and 0.57, respectively (**Table 5**). The calibration curve was drawn with repeated self-sampling, indicating satisfactory predictive performance. At the same time, DCA was performed to evaluate the impact of clinical decisions, which showed the joint prediction model to have better benefits throughout the threshold probability range (**Figure 8**).

**Table 4:** Characteristics of the training set and the validation set.

Characteristics		Training set (right colon)	Validation set (left colon)	*P*
Sex	Male patients	16 (52%)	7 (50%)	0.837
	Female patients	14 (48%)	7 (50%)	
Age	<50 years	7 (24%)	3 (21%)	0.888
	≥50 years	23 (76%)	11 (79%)	
BMI	<18.5	3 (10%)	1 (8%)	0.643
	18.5–24	17 (58%)	10 (71%)	
	>24	10 (32%)	3 (21%)	
Location	Ileocecal	6 (21%)	Descending colon 2 (14%)	0.918
	Colon ascendens	14 (48%)	Rectum 7 (50%)	
	Hepatic flexure	9 (29%)	Sigmoid colon 4 (29%)	
	Transverse colon	1 (2%)	Splenic flexure 1 (7%)	
Long diameter	<5 cm	10 (32%)	5 (36%)	0.877
	≥5 cm	20 (68%)	9 (64%)	
Morphology	Ulcerative type	18 (61%)	10 (71%)	0.463
	Bulge type	12 (39%)	4 (29%)	
	Cauliflower type	0 (0%)	0 (0%)	
	Infiltrating type	0 (0%)	0 (0%)	
Histological type	Without mucus	16 (53%)	7 (50%)	0.837
	Mucus or signet ring cell	14 (47%)	7 (50%)	
pT	T_1_	1 (1%)	0 (0%)	0.825
	T_2_	4 (12%)	3 (21%)	
	T_3_	20 (69%)	9 (63%)	
	T_4_	5 (18%)	2 (14%)	
pN	N_0_	22 (75%)	9 (64%)	0.540
	N_+_	8 (25%)	5 (36%)	
pM	M_0_	27 (90%)	13 (93%)	0.759
	M_1_	3 (10%)	1 (7%)	
pTNM	I	3 (11%)	2 (14%)	0.967
	II	18 (61%)	8 (59%)	
	III	6 (18%)	3 (21%)	
	IV	3 (10%)	1 (7%)	
Intravascular cancer thrombus	No	24 (81%)	11 (79%)	0.913
	Yes	6 (19%)	3 (21%)	
Nerve invasion	No	21 (72%)	9 (64%)	0.705
	Yes	9 (28%)	5 (36%)	
Total		*n* = 30	*n* = 14	
Differentiated^a^	High	0 (0%)	0 (0%)	0.905
	High-medium	1 (4%)	1 (9%)	
	Medium	14 (52%)	6 (55%)	
	Medium-low	6 (22%)	2 (18%)	
	Low	6 (22%)	2 (18%)	
Total		*n* = 27	*n* = 11	

pT, pathological primary tumor; pN, pathological regional lymph node; pM, pathological distant metastasis.

a Missing pathology.

**Table 5:** Receiver operating characteristic (ROC) analysis of the left colon with a joint model under random forest.

Left colon	AUC (95%CI)	Accuracy	Sensitivity	Specificity
Validation set	0.81 (0.77–0.85)	0.70	0.85	0.57

## 4 Discussion

In 2012, Lambin introduced the concept of radiomics, which is defined as the process of extracting large-scale quantitative features from medical images, followed by feature selection and predictive model construction (5–8). Subsequently, Tuan developed and validated a novel CT-based index for pre-radiotherapy assessment of non-small cell lung cancer (9). In recent years, there has been a growing body of research applying radiomics analysis to differentiate benign from malignant colorectal tumors (10, 11). Currently, diagnosis of the dMMR status primarily relies on an invasive biopsy, which poses considerable clinical challenges (e.g., invasiveness, potential complications, and sampling bias). Thus, the development of a noninvasive predictive method for dMMR status holds significant clinical value for guiding treatment decisions. In the present study, we trained and validated an RF-based predictive model as a tool that was expected to be applied for the diagnosis of dMMR in right colon cancer. In addition, we screened the most important clinically relevant variable features from numerous colon cancer-related characteristics. Through these analyses, clinicians can utilize the model established by this algorithm to identify dMMR-positive patients, which is expected to optimize the risk stratification in clinical practice. Model construction involved three key steps: 1) segmentation of the lesion from the CT images, noting that inter-operator subjectivity may compromise the repeatability of the results (12); 2) conversion of image features into numerical data; and 3) application of four ML algorithms. To mitigate delineation variability, interobserver consistency was examined using texture features from 10 randomly selected patients whose lesions were independently outlined by two radiologists. Features with an ICC > 0.75 were retained for subsequent LASSO analysis (13–15). Notably, Liang demonstrated that LASSO-based radiomics could distinguish CRC stages I–II from III–IV with an AUC of 0.792, underscoring its potential for preoperative staging (16).

Radiomics models leveraged ML algorithms to establish optimal feature classification boundaries (17). Currently, there is no consensus on the optimal ML algorithm; however, LR, RF, SVM, and other algorithms have been widely applied in CRC radiomics prediction models, particularly for genotyping, tumor staging, pathological response assessment, and survival analysis (18, 19, 26, 27). Deist compared radiomics models for predicting radiotherapy outcomes and found that the LR and RF models outperformed others (28), whereas conflicting evidence suggests that SVM may yield marginally better results than LR (29–31). In our study, LR, RF, SVM, and XGBoost were employed for radiomics analysis of preoperative primary lesions to maximize diagnostic efficiency. The training set achieved AUC values of 0.91 (LR), 0.986 (RF), 0.944 (SVM), and 0.977 (XGBoost), with corresponding test set AUC values of 0.898, 0.967, 0.917, and 0.945. Notably, the RF algorithm demonstrated superior performance, consistent with its proven utility in predicting histological differentiation in CRC and non-small cell lung cancer (32, 33). However, in the era of big data and precision medicine, single clinical or imaging-based models have increasingly failed to meet individualized needs. Joint models integrating clinical characteristics and radiomics have shown significant optimization, enhancing predictive efficiency (34).

The primary objective of this study was to establish a noninvasive approach for predicting the MMR status using radiomics.

First, we successfully achieved this goal via the RF algorithm and validated the efficacy of this model. Second, we constructed a clinical model incorporating partial postoperative pathological parameters, which also yielded certain predictive performance, but was inferior to the radiomics model. Third, a joint model was developed by combining the RF algorithm with clinical data, and this framework also included postoperative pathological parameters. Currently, the MMR status is predominantly reliant on immunohistochemistry or genetic testing. These tests are typically performed after routine pathological examination, which not only delays the treatment time but also increases the financial burden. In contrast, a radiomics model has already demonstrated favorable predictive efficacy for MMR status; furthermore, the joint model can maximize the predictive performance to the greatest extent. Notably, patients undergoing enhanced recovery after surgery and discharged early may not be guaranteed timely access to the MMR status results.

Previous studies have demonstrated the utility of joint radiomics–clinical models for the prediction of the MMR status. For instance, in a cohort of 119 stage II CRC patients, a joint model achieved an AUC of 0.752 (35), while another study in stage II–III CRC reported moderate predictive performance of a joint model, with AUC values of 0.80 in the training set and 0.79 in the test set (36). Notably, these joint models exhibited superior predictive performance for dMMR in CRC (35–37), which is consistent with our findings that the RF algorithm outperformed previous reports (ACU = 0.978 *vs*. 0.90) (37). In addition, validation using data of the left colon yielded comparable results, indicating that radiomics-based dMMR prediction is generalizable across the entire colon and is not restricted to right-sided lesions. The clinical utility and the reliability of this comprehensive model were further validated using DCA, reinforcing radiomics as an emerging noninvasive tool to assist in clinical decision-making and patient management.

This study has several limitations that warrant discussion. First, as a single-center retrospective analysis, the generalizability of our model may be limited. Prospective multicenter studies with bigger sample sizes are essential to validate and refine these findings. Second, although immunohistochemistry is a widely used and reliable method for assessing the dMMR status, PCR remains the gold standard for definitive MMR, which may have introduced validation bias. Third, the exclusion of patients with incomplete or inconsistent clinical data inevitably introduced selection bias, potentially affecting the representativeness of this cohort. Fourth, despite the performance of interobserver consistency testing, manual delineation of the ROIs introduced inherent subjectivity. The development and the adoption of artificial intelligence-driven automated ROI segmentation technologies are critical for enhancing research reproducibility (38, 39). Finally, external validation via a prospective multicenter trial is necessary to mitigate the inherent biases of retrospective designs and to establish the model’s clinical applicability across diverse populations.

## 5 Conclusion

In this study, a joint predictive model was constructed by incorporating the RF algorithm with clinicopathological variables, and its performance in predicting the dMMR status in right colon cancer was systematically evaluated. The RF algorithm, a powerful ensemble learning method known for its ability to handle high-dimensional data, capture complex nonlinear relationships, resist overfitting through bootstrap aggregation, and feature randomness, was adopted as the core ML framework. By incorporating key clinicopathological features—including age, tumor size, histological grade, and lymph node, among others—the joint model harnesses both the superior pattern recognition capability of the RF algorithm and the clinical relevance of pathological indicators. This integration enables the model to synergistically leverage computational analytics and clinical expertise for enhanced predictive performance.

## Data Availability

The original contributions presented in the study are included in the article/supplementary material. Further inquiries can be directed to the corresponding author.

## References

[1] Fedorov A Beichel R Kalpathy-Cramer J Finet J Fillion-Robin JC Pujol S . 3D Slicer as an image computing platform for the Quantitative Imaging Network. Magn Reson Imaging. (2012) 30:1323–41. doi: 10.1016/j.mri.2012.05.001, PMID: 22770690 PMC3466397

[2] van Griethuysen J Fedorov A Parmar C Hosny A Aucoin N Narayan V . Computational radiomics system to decode the radiographic phenotype. Cancer Res. (2017) 77:e104–7. doi: 10.1158/0008-5472.CAN-17-0339, PMID: 29092951 PMC5672828

[3] Zhao W Yang J Ni B Bi D Sun Y Xu M . Toward automatic prediction of EGFR mutation status in pulmonary adenocarcinoma with 3D deep learning. Cancer Med. (2019) 8:3532–43. doi: 10.1002/cam4.2233, PMID: 31074592 PMC6601587

[4] Vickers AJ Cronin AM Elkin EB Gonen M . Extensions to decision curve analysis, a novel method for evaluating diagnostic tests, prediction models and molecular markers. BMC Med Inform Decis Mak. (2008) 8:53. doi: 10.1186/1472-6947-8-53, PMID: 19036144 PMC2611975

[5] Gillies RJ Kinahan PE Hricak H . Radiomics: images are more than pictures, they are data. Radiology. (2016) 278:563–77. doi: 10.1148/radiol.2015151169, PMID: 26579733 PMC4734157

[6] Lambin P Rios-Velazquez E Leijenaar R . Radiomics: extracting more information from medical images using advanced feature analysis. Eur J Cancer. (2012) 48:441–6. doi: 10.1016/j.ejca.2011.11.036, PMID: 22257792 PMC4533986

[7] Gillies RJ Kinahan PE Hricak H . Radiomics: images are more than pictures, they are data. (2013) 278:563–77. doi: 10.1148/radiol.2015151169, PMID: 26579733 PMC4734157

[8] Aerts HJ Velazquez ER Leijenaar RT Parmar C Grossmann P Carvalho S . Decoding tumour phenotype by noninvasive imaging using a quantitative radiomics approach. Nat Commun. (2014) 5:4006. doi: 10.1038/ncomms5006, PMID: 24892406 PMC4059926

[9] Pham TD Watanabe Y Higuchi M Suzuki H . Texture analysis and synthesis of Malignant and benign mediastinal lymph nodes in patients with lung cancer on computed tomography. Sci Rep. (2017) 7:43209. doi: 10.1038/srep43209, PMID: 28233795 PMC5324097

[10] Yan H Yu TN . Radiomics-clinical nomogram for response to chemotherapy in synchronous liver metastasis of colorectal cancer:Good,but not good enough. World J Gastroenterol. (2022) 28:3. doi: 10.3748/wjg.v28.i9.973, PMID: 35317054 PMC8908283

[11] Badic B Da-Ano R Poirot K Jaouen V Magnin B Gagnière J . Prediction of recurrence after surgery in colorectal cancer patients using radiomics from diagnostic contrast-enhanced computed tomography: a two-center study. Eur radiology. (2022) 1):32. doi: 10.1007/s00330-021-08104-4, PMID: 34170367

[12] Park A Chute C Rajpurkar P Lou J Ball RL Shpanskaya K . Deep learning-assisted diagnosis of cerebral aneurysms using the headXNet model. JAMA Netw Open. (2019) 2:e195600. doi: 10.1001/jamanetworkopen.2019.5600, PMID: 31173130 PMC6563570

[13] Yang L Yang J Zhou X Huang L Zhao W Wang T . Development of a radiomics nomogram based on the 2D and 3D CT features to predict the survival of non-small cell lung cancer patients. Eur Radiol. (2019) 29:2196–206. doi: 10.1007/s00330-018-5770-y, PMID: 30523451

[14] Sun RJ Fang MJ Tang L Li XT Lu QY Dong D . CT-based deep learning radiomics analysis for evaluation of serosa invasion in advanced gastric cancer. Eur J Radiol. (2020) 132:109277. doi: 10.1016/j.ejrad.2020.109277, PMID: 32980726

[15] Sauerbrei W Royston P Binder H . Selection of important variables and determination of functional form for continuous predictors in multivariable model building. Stat Med. (2007) 26:5512–28. doi: 10.1002/sim.3148, PMID: 18058845

[16] Liang C Huang Y He L Chen X Ma Z Dong D . The development and validation of a CT-based radiomics signature for the preoperative discrimination of stage I-II and stage III-IV colorectal cancer. Oncotarget. (2016) 7:31401–12. doi: 10.18632/oncotarget.8919, PMID: 27120787 PMC5058766

[17] Mattonen SA Palma DA Johnson C Louie AV Landis M Rodrigues G . Detection of local cancer recurrence after stereotactic ablative radiation therapy for lung cancer: physician performance versus radiomic assessment. Int J Radiat Oncol Biol Phys. (2016) 94:1121–8. doi: 10.1016/j.ijrobp.2015.12.369, PMID: 26907916

[18] Aoude LG Wong B Bonazzi VF Brosda S Walters SB Koufariotis LT . Radiomics biomarkers correlate with CD8 expression and predict immune signatures in melanoma patients. Mol Cancer Res. (2021) 19:950–6. doi: 10.1158/1541-7786.MCR-20-1038, PMID: 33811161

[19] Li M Zhu YZ Zhang YC Yue YF Yu HP Song B . Radiomics of rectal cancer for predicting distant metastasis and overall survival. World J Gastroenterol. (2020) 26:5008–21. doi: 10.3748/wjg.v26.i33.5008, PMID: 32952346 PMC7476170

[20] Song Z Zou S Zhou W Huang Y Shao L Yuan J . Clinically applicable histopathological diagnosis system for gastric cancer detection using deep learning. Nat Commun. (2020) 11:4294. doi: 10.1038/s41467-020-18147-8, PMID: 32855423 PMC7453200

[21] Kather JN Pearson AT Halama N Jäger D Krause J Loosen SH . Deep learning can predict microsatellite instability directly from histology in gastrointestinal cancer. Nat Med. (2019) 25:1054–6. doi: 10.1038/s41591-019-0462-y, PMID: 31160815 PMC7423299

[22] Park S Pettigrew MF Cha YJ Kim IH Kim M Banerjee I . Deep Gaussian process with uncertainty estimation for microsatellite instability and immunotherapy response prediction from histology. NPJ Digit Med. (2025) 8:294. doi: 10.1038/s41746-025-01580-8, PMID: 40389599 PMC12089473

[23] Yamashita R Long J Longacre T Peng L Berry G Martin B . Deep learning model for the prediction of microsatellite instability in colorectal cancer: a diagnostic study. Lancet Oncol. (2021) 22:132–41. doi: 10.1016/S1470-2045(20)30535-0, PMID: 33387492

[24] Wang CW Muzakky H Lee YC Chung YP Wang YC Yu MH . Interpretable multi-stage attention network to predict cancer subtype, microsatellite instability, TP53 mutation and TMB of endometrial and colorectal cancer. Comput Med Imaging Graph. (2025) 121:102499. doi: 10.1016/j.compmedimag.2025.102499, PMID: 39947084

[25] Feng X Yin W Ye Q Chi Y Wen H Sun Y . Deepath-MSI: a clinic-ready deep learning model for microsatellite instability detection in colorectal cancer using whole-slide imaging. NPJ Precis Oncol. (2025) 9:302. doi: 10.1038/s41698-025-01094-2, PMID: 40877434 PMC12394642

[26] Liu S Yu X Yang S Hu P Hu Y Chen X . Machine learning-based radiomics nomogram for detecting extramural venous invasion in rectal cancer. Front Oncol. (2021) 11:610338. doi: 10.3389/fonc.2021.610338, PMID: 33842316 PMC8033032

[27] Zhang S Yu M Chen D Li P Tang B Li J . Role of MRI−based radiomics in locally advanced rectal cancer (Review). Oncol Rep. (2022) 47:34. doi: 10.3892/or.2021.8245, PMID: 34935061 PMC8717123

[28] Singal AG Mukherjee A Elmunzer BJ Higgins PD Lok AS Zhu J . Machine learning algorithms outperform conventional regression models in predicting development of hepatocellular carcinoma. Am J Gastroenterol. (2013) 108:1723–30. doi: 10.1038/ajg.2013.332, PMID: 24169273 PMC4610387

[29] Zhang YD Wang J Wu CJ Bao ML Li H Wang XN . An imaging-based approach predicts clinical outcomes in prostate cancer through a novel support vector machine classification. Oncotarget. (2016) 7:78140–51. doi: 10.18632/oncotarget.11293, PMID: 27542201 PMC5363650

[30] Zhou Z Folkert M Cannon N Iyengar P Westover K Zhang Y . Predicting distant failure in early stage NSCLC treated with SBRT using clinical parameters. Radiother Oncol. (2016) 119:501–4. doi: 10.1016/j.radonc.2016.04.029, PMID: 27156652 PMC4930894

[31] Wu HY Gong C Lin SP Chang KY Tsou MY Ting CK . Predicting postoperative vomiting among orthopedic patients receiving patient-controlled epidural analgesia using SVM and LR. Rep. (2016) 6:27041. doi: 10.1038/srep27041, PMID: 27247165 PMC4887988

[32] Chen X Fang M Dong D Wei X Liu L Xu X . A radiomics signature in preoperative predicting degree of tumor differentiation in patients with non-small cell lung cancer. Acad Radiol. (2018) 25:1548–55. doi: 10.1016/j.acra.2018.02.019, PMID: 29572049

[33] Huang X Cheng Z Huang Y Liang C He L Ma Z . CT-based radiomics signature to discriminate high-grade from low-grade colorectal adenocarcinoma. Acad Radiol. (2018) 25:1285–97. doi: 10.1016/j.acra.2018.01.020, PMID: 29503175

[34] Huang Y Liu Z He L Chen X Pan D Ma Z . Radiomics signature: A potential biomarker for the prediction of disease-free survival in early-stage (I or II) non-small cell lung cancer. Radiology. (2016) 281:947–57. doi: 10.1148/radiol.2016152234, PMID: 27347764

[35] Fan S Li X Cui X Zheng L Ren X Ma W . Computed tomography-based radiomic features could potentially predict microsatellite instability status in stage II colorectal cancer: A preliminary study. Acad Radiol. (2019) 26:1633–40. doi: 10.1016/j.acra.2019.02.009, PMID: 30929999

[36] Golia Pernicka JS Gagniere J Chakraborty J Yamashita R Nardo L Creasy JM . Radiomics-based prediction of microsatellite instability in colorectal cancer at initial computed tomography evaluation. Abdom Radiol (NY). (2019) 44:3755–63. doi: 10.1007/s00261-019-02117-w, PMID: 31250180 PMC6824954

[37] Wu J Zhang Q Zhao Y Liu Y Chen A Li X . Radiomics analysis of iodine-based material decomposition images with dual-energy computed tomography imaging for preoperatively predicting microsatellite instability status in colorectal cancer. Front Oncol. (2019) 9:1250. doi: 10.3389/fonc.2019.01250, PMID: 31824843 PMC6883423

[38] Jian J Xiong F Xia W Zhang R Gu J Wu X . Fully convolutional networks (FCNs)-based segmentation method for colorectal tumors on T2-weighted magnetic resonance images. Australas Phys Eng Sci Med. (2018) 41:393–401. doi: 10.1007/s13246-018-0636-9, PMID: 29654521

[39] Huang L Xia W Zhang B Qiu B Gao X . MSFCN-multiple supervised fully convolutional networks for the osteosarcoma segmentation of CT images. Comput Methods Programs Biomed. (2017) 143:67–74. doi: 10.1016/j.cmpb.2017.02.013, PMID: 28391820

